# Electrochemical Reduction Pathways from Goethite to Green Iron in Alkaline Solution with Silicate Additive

**DOI:** 10.1021/acssuschemeng.4c08451

**Published:** 2025-02-11

**Authors:** Divakar Arumugam, Tongxin Zhou, Sathya Narayanan Jagadeesan, Ranga Teja Pidathala, Lihua Zhang, AM Milinda Abeykoon, Gihan Kwon, Daniel Olds, Badri Narayanan, Xiaowei Teng

**Affiliations:** † Department of Chemical Engineering, 8718 Worcester Polytechnic Institute, 100 Institute Road, Worcester, Massachusetts 01609, United States; ‡ Department of Mechanical Engineering, 5170 University of Louisville, 332 Eastern Pkwy, Louisville, Kentucky 40292, United States; § National Synchrotron Light Source II, 8099 Brookhaven National Laboratory, Upton, New York 11973, United States; ∥ National Synchrotron Light Source II, 8099 Brookhaven National Laboratory, 743 Brookhaven Avenue, Upton, New York 11973, United States

**Keywords:** Iron Electrolysis, Zero-emission, FeOOH, Silicate, Hydrogen evolution reaction

## Abstract

Energy-efficient and low-temperature iron electrolysis in alkaline solutions is a low-cost and sustainable ironmaking process with zero-carbon emissions when renewable electrical sources are involved. However, its implementation is hindered by electrochemically inert Fe_3_O_4_ and parasitic H_2_ gas formation during the electrochemical reduction process, resulting in the low energy efficiency of iron electrolysis. Here, we further explore the potential of electrochemical reduction of goethite (FeOOH) by employing a low concentration of silicate additive in an alkaline solution to mitigate Fe_3_O_4_ accumulation and H_2_ generation. Electrochemical measurements coupled with operando X-ray diffraction and X-ray absorption spectroscopy suggested FeOOH → Fe_3_O_4_ → Fe­(OH)_2_ → Fe reduction pathways. Interestingly, a poorly crystalline or amorphous Fe­(OH)_2_ phase formed in the NaOH/silicate mixed electrolyte, possibly due to the inhibitive effect of silicate on water and ion transport, which eventually contributed to the improved reduction of Fe_3_O_4_, also supported by atomistic simulations. This work demonstrates the potential for silicate as a low-cost and effective electrolyte additive to improve room-temperature green iron formation via electrolysis.

## Graphical abstract

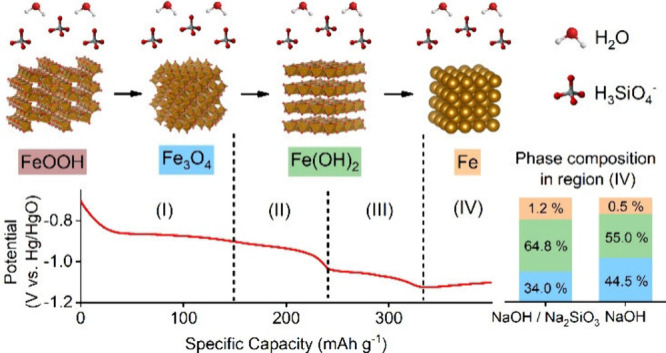

## Introduction

CO_2_ emissions have become one of the most critical environmental concerns. The Intergovernmental Panel on Climate Change Assessment concluded that reaching the goal of limiting global warming to 1.5 °C by 2050 requires rapid transitions to low-carbon emissions in all sectors.^1,2^ Ironmaking contributes the most substantial CO_2_ emissions in the industrial sectors, responsible for ∼8% of all anthropogenic CO_2_ emissions globally. The industrial ironmaking process requires high-temperature operations and carbon as a reducing agent that releases CO_2_ as a byproduct.^3–5^ Considering the rapid rise in global steel production, CO_2_ emissions from ironmaking should radically decrease to meet the 2050 challenge.^6,7^

Two potential ways to separate iron from oxygen without CO_2_ emission entail either H_2_ or electrical energy to reduce iron ore to iron metal (Fe^3+^ → Fe^0^).^3^ Hydrogen-based direct reduction (HDR) has been investigated as a low-emission steelmaking technology.^8,9^ However, it requires a high equilibrium H_2_ concentration and additional H_2_ separation cycles for better H_2_ utilization,^8^ significantly increasing system complexity and production costs. Catering to the industrial demands of HDR also requires infrastructures for H_2_ production, storage, and transportation, imposing another challenge for its implementation.^10^ Electrochemical ironmaking, so-called iron electrolysis, includes molten oxide electrolysis (MOE) and aqueous electrowinning, offering zero-carbon emissions when using renewable sources to produce chemically pure Fe and steel by consecutive electrochemical alloying processes.^11–13^ MOE separates liquid iron from oxygen at operating temperatures above the melting point of iron (1538 °C). This high-temperature MOE has several technical challenges, especially the lack of a corrosive-resistant anode to oxidize lattice oxygen ions to the oxygen species, O_2_. Although noble metal-based anode materials (e.g., iridium) are promising, their scarcity and high cost make large-scale implementation of MOE unlikely.^14^

Aqueous electrochemical reduction of iron is traditionally operated by the so-called “electrowinning process” at low temperatures in alkaline solutions via a two-step procedure involving the dissolution of the iron ore into Fe^2+^ and the electrodeposition of Fe^2+^ into metallic Fe.^13,15,16^ While the dissolution–deposition process remains an active research area, the limited contact between the liquid and the electrode, corresponding to liquid-to-surface mass-transfer control, poses a main threshold for electrolysis productivity.^17,18^ Alternatively, all-solid-state electrochemical reduction, where the reduction of iron oxide to iron metal only involves solid-phase iron species by bypassing dissolution, shows great promise for large-scale development.^19^ However, most of the reported iron electrolysis studies are conducted in concentrated alkaline solution (pH >14), resulting in the inevitable formation of soluble HFeO_2_^–^ species.^20^ The lack of knowledge of reduction mechanisms hinders the all-solid-state iron electrolysis at a moderate alkaline solution (pH <13), and there is no structural evidence to support the electrochemical reduction pathways. Therefore, a more precise understanding is required to implement the solid-phase electrochemical formation of iron in low-temperature weak alkaline solutions. Previous studies on iron alkaline batteries also suggested that the formation of electrochemically inert Fe_3_O_4_ and parasitic H_2_ gas via the hydrogen evolution reaction (HER) poses challenges to Fe redox, resulting in incomplete Fe redox and energy loss. This work shows that weak NaOH solution (pH 12) combined with low-concentration sodium silicate (Na_2_SiO_3_) additive (5000 ppm) can effectively reduce water activity and ion transport to mitigate HER effectively and promote Fe_3_O_4_ reduction conversion by facilitating solid-state ionic transport, allowing the FeOOH → Fe_3_O_4_→ Fe­(OH)_2_ → Fe reaction to proceed with improved formation rates for Fe­(OH)_2_ and Fe while minimizing Fe_3_O_4_ accumulation (Figure 1). FeOOH was chosen as the model material for this study because it is the thermodynamically most stable high-valence iron species in an aqueous environment and is also the common form of iron oxide in the weathering of primary iron minerals and iron ore deposits.

**Figure 1:**
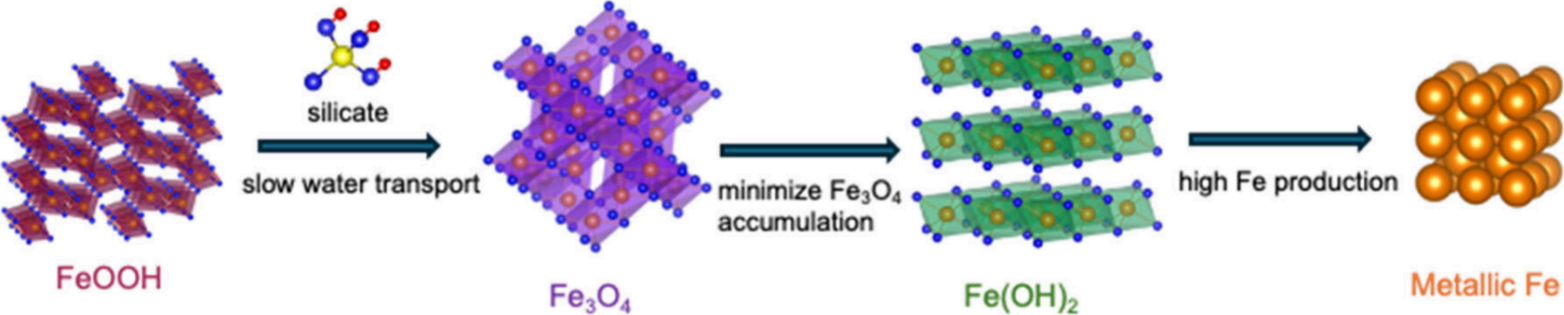
Schematics of electrochemical reduction pathways of FeOOH. The silicate additive help slow water transport, minimize Fe_3_O_4_ accumulation, and promote Fe formation.

## Experimental Section

### Material and Equipment

The goethite (FeOOH) material was synthesized by using the following procedures. Ferrous sulfate heptahydrate (FeSO_4_·7H_2_O, 2.085 g) and ferric sulfate (Fe_2_(SO_4_)_3_·*x*H_2_O, 0.66 g) were dissolved in 50 mL of deionized (DI) water. NaOH (0.6 g) dissolved in 50 mL of DI water was added to the mixture of iron salts. The solution was stirred for 24 h under air overflow. The obtained product was washed and dried overnight in an open atmosphere and ground for electrochemical characterizations. All chemicals are high-purity and were obtained from Alfa Aesar, Inc.

High-resolution transmission electron microscopy (HRTEM) and high-angle annular dark-field (HAADF) imaging were conducted at the Center for Functional Nanomaterials in Brookhaven National Laboratory (BNL), using an FEI Talos F200x Scanning TEM system.

Operando X-ray diffraction (XRD) and X-ray absorption spectroscopy (XAS) measurements were done at Beamlines 28-ID-1 and 6-BM at the National Synchrotron Light Source-II at BNL. Details of the operando measurements test are provided in the Supporting Information.

#### Half-Cell Electrochemical Studies

Chronopotentiometry (CP) measurements were conducted in a CH Instruments 660D/E electrochemical workstation using a three-electrode half-cell system for Tafel and pulse-relaxation electrochemical analyses. Details of the half-cell tests are provided in the Supporting Information.

#### Reactive Molecular Dynamics Simulations (RMD) and Density Functional Theory (DFT) Calculations

RMD simulations were performed using the widely recognized open-source package LAMMPS.^21–23^ DFT calculations were performed within the framework of Hubbard-corrected DFT theory (DFT + *U*), using the projected augmented plane wave method as implemented in VASP.^24–26^ Details of RMD and DFT calculations are provided in the Supporting Information.

## Results and Discussion

### Synthesis of Goethite Materials

The present work investigates the feasibility of the all-solid-state electrochemical reduction of FeOOH in a dilute NaOH solution at room temperature. As shown in Figure S1, α-FeOOH (goethite) powder was coated on carbon paper and then immersed in a NaOH solution (pH 12). A direct current was applied between the goethite anode and the platinum wire cathode. FeOOH was synthesized using the coprecipitation method using a fed-batch reactor, where ferric sulfate reacts with NaOH solution injected via a separate stream. After the solution-phase synthesis, the collected materials were thermally treated in the open air. XRD of the final product, as shown in Figure 2a, confirms the formation of pure phase α-FeOOH. The high-resolution TEM (HR-TEM) image in Figure 2b–e showed the nanorod morphology of the FeOOH with an average width of ∼10 nm and length of ∼70 nm. The FeOOH is highly crystalline with distinct interlayer spacings of 4.13 and 2.65 Å, corresponding to (110) and (130) diffraction planes, respectively.

**Figure 2:**
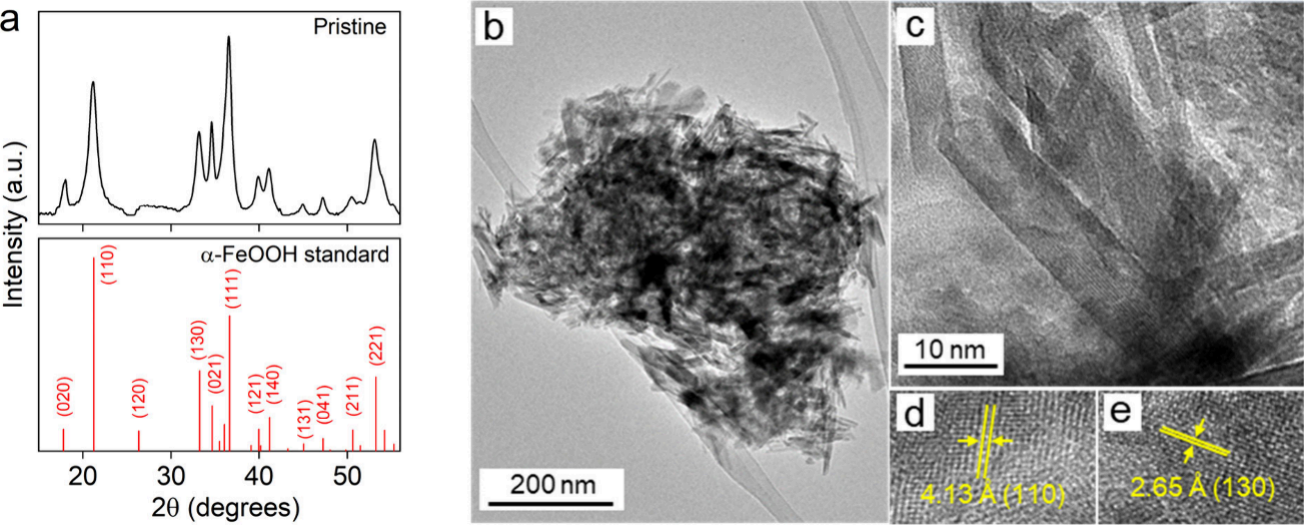
Structural characterization of FeOOH: (a) XRD of FeOOH and (b–e) high-resolution TEM images of FeOOH (panels (d) and (e) show well-defined crystalline fringes).

### Operando XRD To Analyze the Structural Evolution during the Reduction

An iron electrolysis cell was constructed and tested in a three-electrode cell setting by using a FeOOH anode, a commercial Pt wire cathode, and a Ag/AgCl reference electrode. The electrolyte volume was 5 mL at room temperature under atmospheric pressure (see Figure S1 for the electrolysis cell design). Operando XRD studies the structural evolution of the FeOOH anode during iron electrolysis in two-electrolyte systems, including 0.01 M NaOH/5000 ppm silicate and 0.01 M NaOH-only solutions. The measurements were conducted in a three-electrode half-cell using CP measurements. The contour plots of operando XRD are presented in Figure 3, and the stacked waterfall plots of XRD patterns are shown in Figure S2. Figure 3 also shows the evolution of molar ratios of various iron forms, including FeOOH, Fe_3_O_4_, Fe­(OH)_2_, and Fe, obtained from Rietveld refinements based on total Fe atoms, as summarized in Table 1.

**Figure 3:**
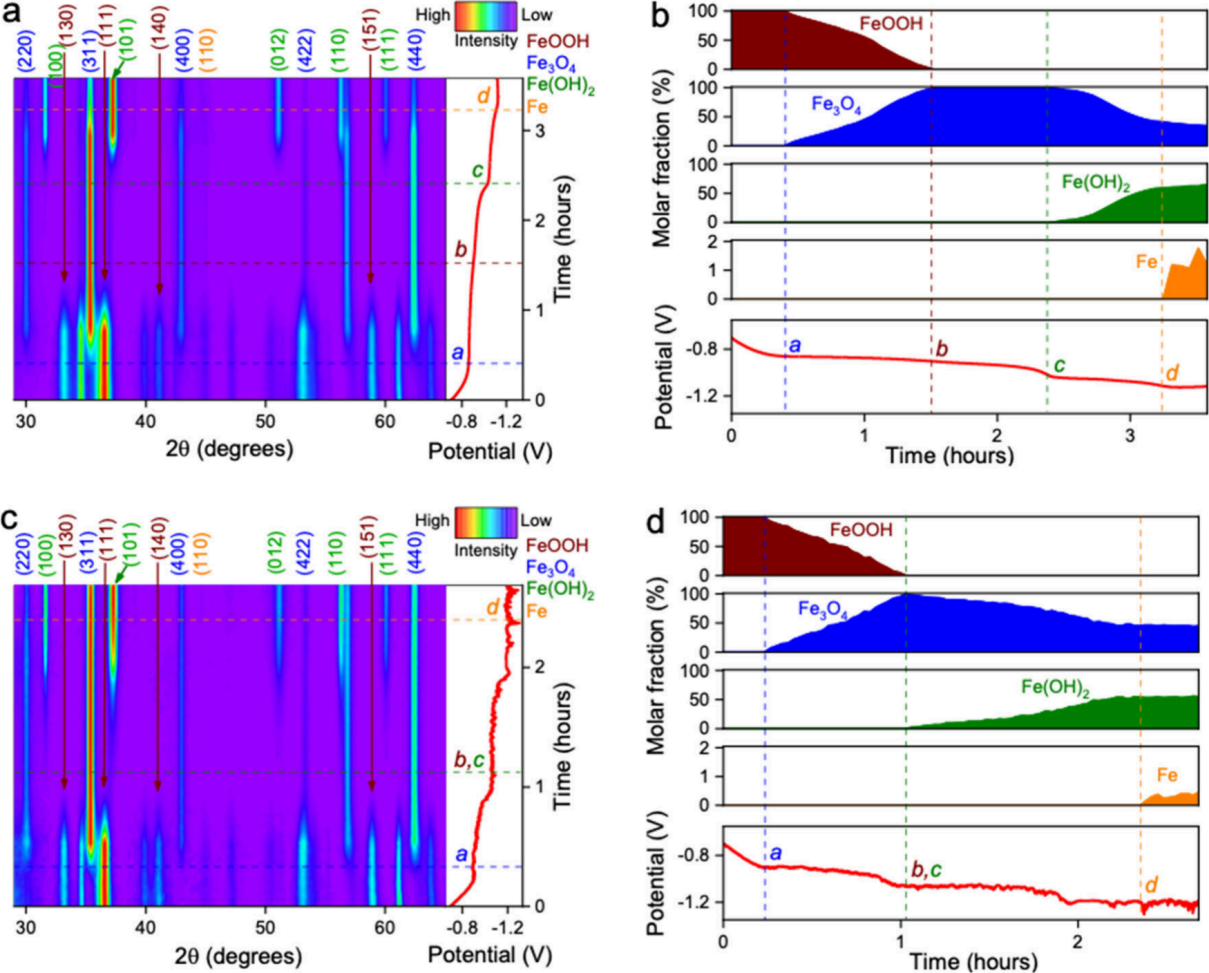
Contour plots obtained from operando XRD and the molar fraction of phases based on total Fe atoms calculated by Rietveld refinement in (a, b) NaOH/Na_2_SiO_3_ and (c, d) NaOH electrolytes, acquired simultaneously with CP measurements at a current density of 0.1 A g^–1^ plotted with potential vs Hg/HgO. Note: Labels *a*, *c*, and *d* inside the figures mark the start points of Fe_3_O_4_, Fe­(OH)_2_, and Fe formation, and label *b* inside the figures marks the complete consumption of FeOOH, respectively.

**Table 1:** Summaries of the Molar Ratio of FeOOH, Fe_3_O_4_, Fe­(OH)_2_, and Fe during FeOOH Reduction Obtained from Operando XRD Rietveld Refinements

		Molar Ratio (mol %)			
electrolytes/points (potential)		FeOOH	Fe_3_O_4_	Fe(OH)_2_	Fe
NaOH and silicate	*a* (−0.86 V)	100	0	0	0
	*b* (−0.9 V)	0	100	0	0
	*c* (−1.04 V)	0	100	0	0
	*d* (−1.1 V)	0	41.1	58.9	0
	end point (−1.12 V)	0	34.0	64.8	1.2
					
NaOH	*a* (−0.90 V)	100	0	0	0
	*b*/*c* (−1.06 V)	0	100	0	0
	*d* (−1.18 V)	0	45.8	54.2	0
	end point (−1.2 V)	0	44.5	55.0	0.5

Figures 3a and 3b show the FeOOH reduction in the solution of 0.01 M NaOH/5000 ppm silicate from −0.7 V to −1.2 V (vs Hg/HgO), following FeOOH → Fe_3_O_4_ → Fe­(OH)_2_ → Fe reduction pathways. Specifically, the reduction of FeOOH to Fe_3_O_4_ (JCPDS File No. 19–0629) started when the potential decreased to −0.86 V (point *a*) and completed when the potential reached −0.90 V (point *b*) with an average Fe_3_O_4_ formation rate of 1.57 mol % per minute (based on the molar ratio of Fe from Fe_3_O_4_), following eq 1. The Fe­(OH)_2_ (JCPDS File No. 13–0089) starts to form when the potential reaches −1.04 V (Point *c*) with signature Fe­(OH)_2_ diffraction peaks of (001), (011), and (102), following eq 2. As the potential decreased from −1.0 V to −1.1 V, the Fe­(OH)_2_ ratio increased rapidly to 57.9%, with an average growth rate of 1.45 mol % per minute. Metallic Fe forms when the potential decreases to −1.1 V (point *d*), at which Fe_3_O_4_ and Fe­(OH)_2_ still exist with molar ratios of ∼41.1% and 58.9%, respectively. However, it is highly likely that the Fe forms upon Fe­(OH)_2_ reduction in an alkaline solution (eq 3) instead of a direct Fe_3_O_4_ → Fe pathway, because Fe_3_O_4_/Fe redox usually occurs in anhydrous electrochemical conditions.^27^ Notably, metallic Fe formation has slower electrokinetics than Fe_3_O_4_ and Fe­(OH)_2_ (Table 2). It increases to 1.2% when the potential decreases from −1.1 V to −1.12 V throughout 20 min, corresponding to a growth rate of 0.059 mol % per min. The Faradaic efficiency of the FeOOH → Fe_3_O_4_ → Fe­(OH)_2_ → Fe electrochemical reduction process is calculated to be 76% in NaOH/silicate electrolytes by measuring the portion of the total electrical charge used to achieve the experimentally measured mixture of Fe_3_O_4_ (34.0%), Fe­(OH)_2_ (64.8%), and Fe (1.2%). The electrical energy needed to produce such a mixture is calculated to be 1.1 MJ per kg. This value corresponds to an estimated energy consumption of 6.4 GJ per ton of Fe production, assuming the complete FeOOH → Fe conversion without H_2_ gas generation.

1 $$3\mathrm{FeOOH}+e^{-}\to {\mathrm{Fe}}_{3}O_{4}+{\mathrm{OH}}^{-}+H_{2}O$$

2 $${\mathrm{Fe}}_{3}O_{4}+4H_{2}O+2e^{-}\to 3\mathrm{Fe}{\left(\mathrm{OH}\right)}_{2}+2{\mathrm{OH}}^{-}$$

3 $$\mathrm{Fe}{\left(\mathrm{OH}\right)}_{2}+2e^{-}\to \mathrm{Fe}+2{\mathrm{OH}}^{-}$$

**Table 2:** Summaries of Formation Kinetics of Fe_3_O_4_, Fe­(OH)_2_, and Fe during FeOOH Reduction

	Fe_3_O_4_		Fe(OH)_2_		Fe	
electrolyte	formation rate (mol % min^–1^)	max. ratio	formation rate (mol % min^–1^)	max. ratio	formation rate (mol % min^–1^)	max. ratio
NaOH/silicate	1.57	100	1.45	64.8	0.059	1.2
NaOH	2.00	100	0.722	55.0	0.023	0.5

Figures 3c and 3d show the operando XRD of FeOOH reduction in NaOH, following the same reduction pathway of FeOOH → Fe_3_O_4_ → Fe­(OH)_2_ → Fe. Unlike the reduction in NaOH/silicate solution, the formation of Fe_3_O_4_ occurs at a fast rate in NaOH. Still, Fe­(OH)_2_ and Fe formation rates in NaOH solution decreased significantly. Although FeOOH was also entirely reduced to Fe_3_O_4_ in NaOH, the reduction of Fe_3_O_4_ to Fe­(OH)_2_ showed a very low conversion: 44.5% of total Fe atoms remained in Fe_3_O_4_ phases even when metallic Fe started to form. The inert nature of Fe_3_O_4_ poses significant barriers to Fe­(OH)_2_ formation, which consequentially constrains Fe formation. As a result, the final Fe phase ratio reaches only ∼0.5% of total Fe atoms, with a marginal Fe formation rate of 0.023 mol % per minute.

### Operando X-ray Absorption Spectroscopy (XAS) To Analyze the Fe Valence Evolution

It is worth mentioning that the molar ratio of the Fe_3_O_4_ crystalline phase from operando XRD remained unchanged (100%) when the potential decreased from −0.90 V to −1.04 V (between point *b* and point *c* in Figure 3). Such potential range in CP measurements corresponds to a reduction capacity of 90 mAh/g (∼0.9 h), suggesting that faradaic behaviors do not belong to crystalline phases. Operando XAS measurements were then performed to elucidate the valence evolution of entire Fe species, including crystalline and amorphous phases, in contrast to XRD, which only analyzes Bragg diffraction and cannot detect possible amorphous iron oxide or hydroxide formation. Still, amorphous phase contribution to Fe valence evolution was detectable by XAS.^28,29^ The time-resolved XAS experiments were performed using the same setup as operando XRD.

Figure 4a displays a series of Fe K-edge spectra measured during the electrochemical reduction of FeOOH in a NaOH/silicate solution. Figure 4c shows the corresponding contour plot to further elucidate XAS line shape evolution. The steady decrease of the energy position at half absorption (1/2 μ_(E)_) was observed throughout the entire electrochemical process, corresponding to a continuous reduction of iron species. The Fourier transform (FT) of the absorption data (R-space data) is shown in Figure 4b, and the corresponding contour plot is shown in Figure 4d. Two main peaks observed in Figure 4b are the Fe–O and Fe–Fe pair from the first two coordination shells. The steady decrease of Fe–O radial distance throughout the electrochemical process is congruent with the reduction of iron species. Consistent with operando XRD, Figure 3 shows that Fe_3_O_4_ started to form at point *a*′ and reached the highest Fe–Fe bond intensity at point *b*′ (suggesting a maximum Fe_3_O_4_ phase ratio at point *b*′); potential drops were observed at *Point c’* and *Point d’*, suggesting Fe­(OH)_2_ and Fe started to form, respectively.

**Figure 4:**
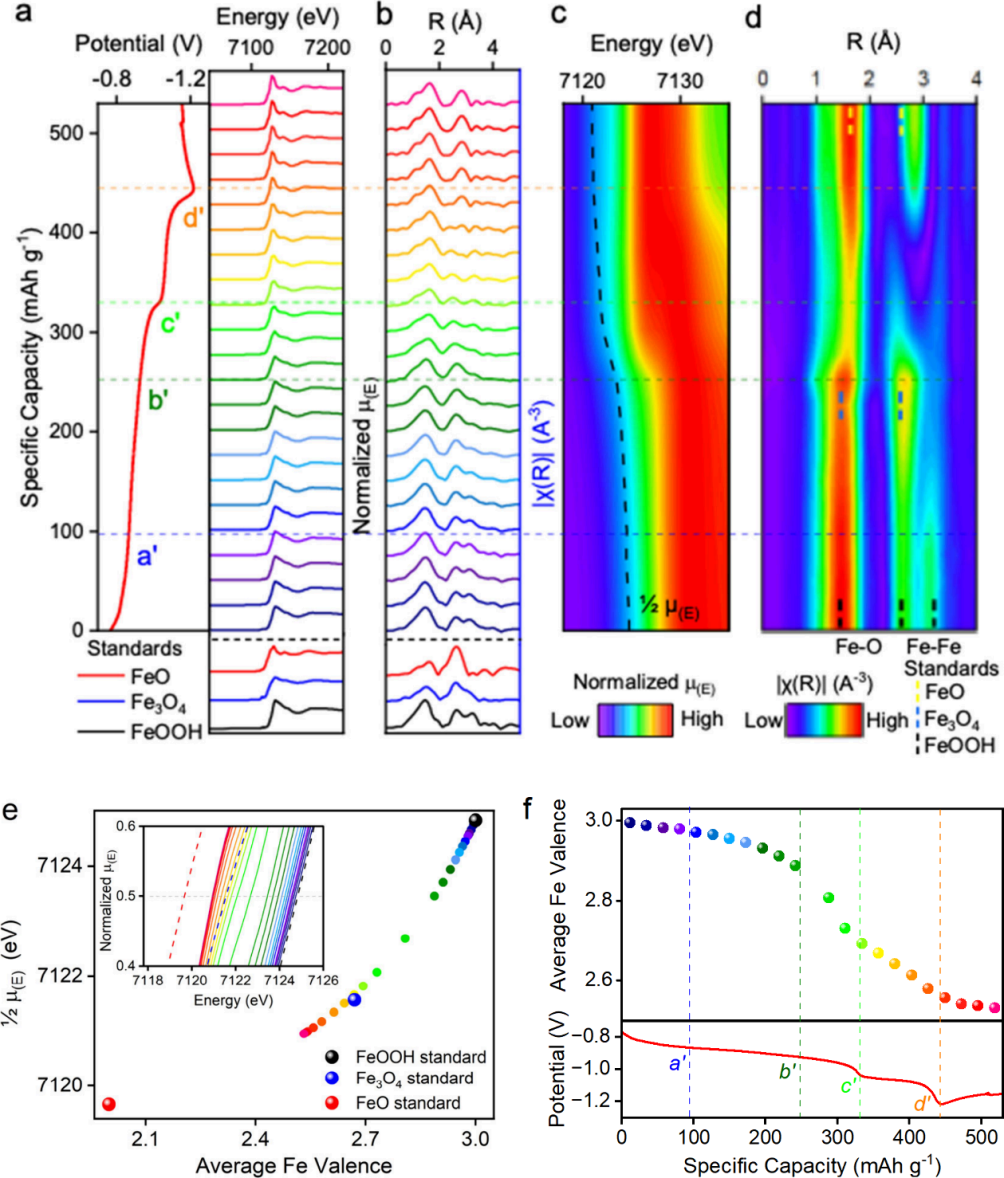
*Operando* XAS measurements were conducted in NaOH/Na_2_SiO_3_ electrolyte. Waterfall plots of (a) Fe–K edge XAS spectra in energy-space acquired simultaneously with CP measurements at a current density of 0.2 A g^–1^ plotted with potential vs Hg/HgO, (b) FT of the XAS spectra in R-space. Contour plots to show the evolution of (c) XAS line shape and 1/2 μ_(E)_ in energy space, (d) Fe–O and Fe–Fe bond distance evolution. (e) Fe valence evolution related to energy shift at 1/2 μ_(E)_ compared with reference iron samples with defined valences. Zoom-in XAS spectra at 1/2 μ_(E)_ are shown in the inset. (f) Evolution of Fe valence during the electrochemical reduction process.

By fitting 1/2 μ_(E)_ energy position with FeO, Fe_3_O_4_, and FeOOH reference compounds with known valence states, the valence evolution of iron species was determined and is plotted in Figures 4e and 4f. Notably, the FeO reference material was used to calibrate the Fe^2+^ valence due to its stable chemical nature, in contrast to the Fe­(OH)_2_ reference, which is prone to oxidizing into Fe_3_O_4_. Interestingly, Figure 4f shows that the Fe valences decreased from +2.88 to +2.69 when the potential decreased from −0.93 V to −1.04 V (between point *b*′ and point *c*′), although the Fe_3_O_4_ crystalline phase ratio remained 100% during the operando XRD measurement (Figure 4b). The discrepancies between operando XRD and XAS suggest the formation of poorly crystalline or amorphous Fe­(OH)_2_ between −0.93 V to −1.04 V, undetected by operando XRD but captured by operando XAS. The FT of the operando XAS data in R-space and the corresponding contour plots (Figures 4b and 4d) also confirmed the formation of poorly crystalline Fe­(OH)_2_, where the intensities of Fe–O and Fe–Fe radial distance decreased greatly between point *b*′ and point *c*′ after the start of Fe_3_O_4_ reduction to Fe­(OH)_2_. While further study is needed, hydrogen codeposition might account for Fe_3_O_4_ reduction to poorly crystalline Fe­(OH)_2_, which is well studied in the electrodeposition of chromium or iron species, where the hydrogen absorption occurs in the metal substrate metal as H atoms (not H_2_ molecules).^30^

### Structural Analysis of the Product

The product after electrochemical reduction was also analyzed by XRD, HR-TEM, and scanning TEM energy-dispersive X-ray spectroscopy (STEM-EDS) mapping. Figure S3 shows the XRD patterns of the final products obtained from the operando electrochemical measurement. All of the diffraction peaks can be indexed to Fe_3_O_4_, Fe­(OH)_2_, and Fe, where the Fe­(OH)_2_ phase ratio in the product prepared in NaOH/silicate electrolytes is higher than that in the NaOH electrolyte (64.8% versus 55%). Figure S4 shows the HR-TEM images of the products prepared from both electrolytes. Highly crystalline Fe_3_O_4_ with large particle sizes were observed, showing distinct interlayer spacings of ∼3.0 Å, corresponding to (220) diffraction plane. The Fe­(OH)_2_ and Fe phases were not observed in HR-TEM, possibly due to the oxidation in the atmosphere environments during the sample preparation. The elemental mapping of Fe, O, and combined Fe and O was presented in Figure S5 to visualize the compositional homogeneities in the final product, where uniformly distributed Fe and O are congruent with XRD and HR-TEM results.

### Mechanism of Promotional Role of Silicate on FeOOH Reduction via Integrated Computational and Experimental Studies

We found that silicate additives influence the transport behavior of the electrochemical system. The silicate (SiO_3_^2–^) anions present in NaOH/Na_2_SiO_3_ aqueous solutions are known to combine with OH^–^ and H^+^ ions to form Si­(OH)_4_ molecules and H_*x*_SiO_4_^(4‑*x*)–^ anions, even at low silicate concentrations, as elucidated by our recent reactive molecular dynamics (RMD) simulations.^31^ Consistent with these reports, our RMD simulations show that SiO_3_^2–^ anions in 0.01 M NaOH with 5000 ppm of Na_2_SiO_3_ exist in the form of Si­(OH)_4_ molecules (∼81%) and H_3_SiO_4_^–^ (∼19%) (Figure 5a). A similar distribution of silicate anions is also observed for 2500 ppm of Na_2_SiO_3_ solutions. Importantly, DFT calculations using the well-established computational hydrogen electrode indicate that H_3_SiO_4_^–^ anions (even in small amounts) can suppress HER on iron surfaces by rendering the hydroxyl desorption significantly more endothermic (via hydrogen bonding) than that on bare Fe.^31^ Indeed, the experimentally observed Tafel slope is the highest (380 mV dec^–1^) in the 0.01 M NaOH/5000 ppm of Na_2_SiO_3_ solution and the lowest (227 mV dec^–1^) in the absence of any silicate additive (Figure S6a); suggesting silicate additives effectively inhibit HER electrokinetics, congruent with DFT calculations. Figure S6b shows the current–pulse relaxation method used to examine silicate’s influence on ion transport, where the chemical diffusion coefficient of the mobile ions is calculated (see the Experimental Methods section in the Supporting Information for more details), and the normalized diffusion coefficient of mobile ions is shown in Figure S6c. The diffusion coefficients of mobile ions in 5000 and 2500 ppm silicate solutions are only 16% and 19% of that in the NaOH-only electrolyte, suggesting that silicate strongly interacts with iron materials, slows the transport of mobile ions and water molecules, and thus imposes high overpotential for electrochemical processes, especially HER. This finding is consistent with our previous RMD simulations, which indicate that silicate anions impede molecular transport by strengthening the hydrogen bond network in water.^31^ Therefore, more electrical energy is used to reduce FeOOH instead of H_2_O, resulting in the observed high conversion of FeOOH → Fe_3_O_4_ → Fe­(OH)_2_ → Fe redox in a NaOH/silicate solution (Table 1).

**Figure 5:**
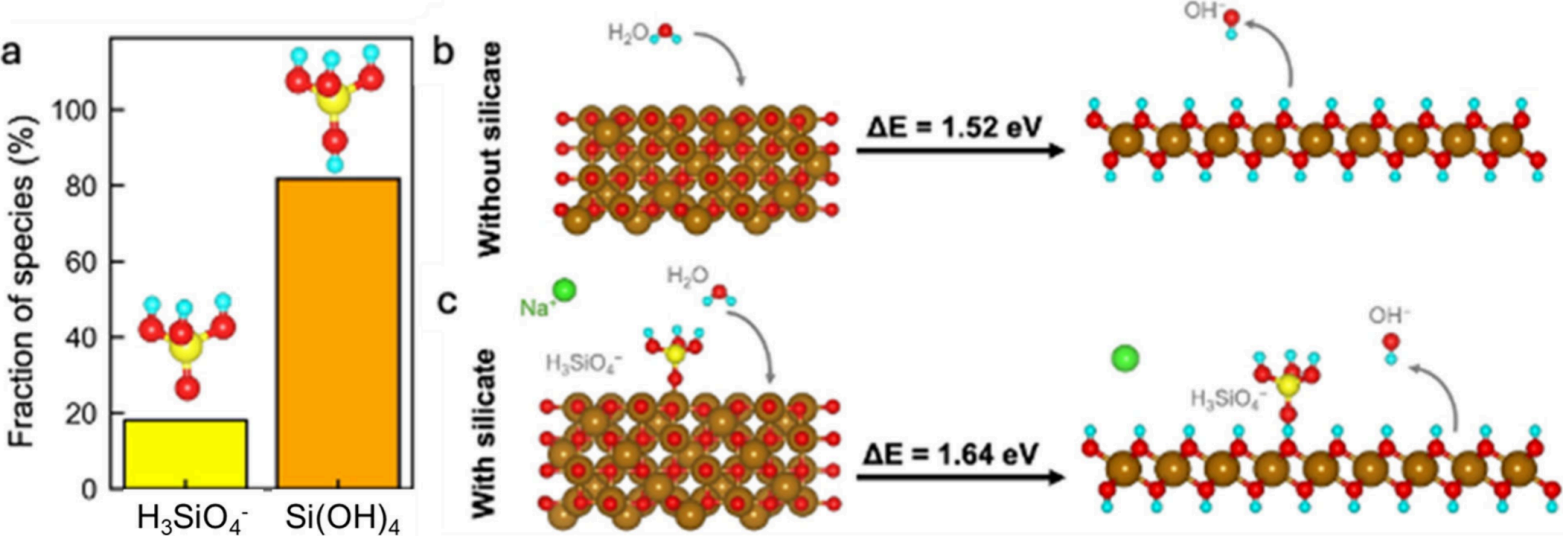
(a) Distribution of silicate species in 0.01 M NaOH aqueous solution with 5000 ppm of Na_2_SiO_3_ additive obtained from RMD. The fraction of silicate anions that exist as Si­(OH)_4_ and H_3_SiO_4_^–^ are shown. The population of each species is averaged over the last 1 ns of representative RMD trajectory. DFT calculation shows energetics of the conversion reaction Fe_3_O_4_ + 4H_2_O + 2e^–^ → 3Fe­(OH)_2_ + 2OH^–^ for (b) without any silicate and (c) with silicate (H_3_SiO_4_^–^) adsorbed. Fe, O, Si, H, and Na atoms are depicted in tan, red, yellow, aqua, and green, respectively.

Silicate additives also greatly influence Fe redox behavior. DFT calculations show that H_3_SiO_4_^–^ anions adsorb strongly on Fe_3_O_4_ and Fe­(OH)_2_ surfaces (adsorption energy on Fe_3_O_4_: – 3.13 eV; Fe­(OH)_2_: – 1.35 eV. Interestingly, the Fe_3_O_4_ → Fe­(OH)_2_ conversion is energetically more uphill (by ∼0.12 eV per unit Fe_3_O_4_) in the presence of adsorbed H_3_SiO_4_^–^ (Figures 5b and 5c). This inhibitive nature of silicate on Fe_3_O_4_ → Fe­(OH)_2_ might explain the formation of poorly crystalline Fe­(OH)_2_ (between points *b* and *c* in Figure 3b and between points *b*′ and *c*′ in Figures 4c and 4d). Notably, the shrinking core mass-transfer model has been used to describe the reduction of iron oxide, considering that the diffusion of oxygenous anions through Fe atoms was the rate-determining step in the electrochemical reduction process. Thus, poorly crystalline or disordered materials have a modified local environment for the Fe–O polyhedra and provide a lower percolation threshold of ion transport in the solid state so that ions (O and Fe) can diffuse easily without directly contacting the Fe–O framework on the atomic scale.^32,33^ Therefore, it is reasonable that forming poorly crystalline or disordered Fe­(OH)_2_ in NaOH/silicate electrolytes improves the solid-state ionic transport to facilitate the Fe_3_O_4_ → Fe­(OH)_2_ → Fe reduction reactions.

In summary, integrated electrochemical measurements, operando X-ray analyses, and atomistic simulation show that the silicate additive inhibits the ions and water transport in the electrolytes to limit HER. It also encourages the formation of poorly crystalline Fe­(OH)_2_ to facilitate the ion transport within the electrode in the solid state. Such two-pronged effects of silicate promote FeOOH → Fe_3_O_4_ → Fe­(OH)_2_ → Fe pathways with minimized Fe_3_O_4_ accumulation and H_2_ formation. Our results offer great promise for iron electrolysis under sustainable conditions at low temperatures and weak alkaline solutions and provide a leap forward in understanding the development of a new industrial process that copes with the environmental issue of greenhouse gas emissions.

## Supplementary Material



## Notes

Detailed information on the operando XRD and XAS measurements; electrochemical measurements; XRD, HR-TEM, STEM-EDS elemental mapping of the final products; and RMD and DFT calculations (Figures S1–S6) (PDF)
