# Supplementary material for: Electrochemical Reduction Pathways from Goethite to Green Iron in Alkaline Solution with Silicate Additive
Source: ACS Sustain Chem Eng. 2025 Feb 11;13(7):2633–40. doi: 10.1021/acssuschemeng.4c08451 (PMC12308757; doi:10.1021/acssuschemeng.4c08451)
Supplement: Supplementary file 1 [file sc4c08451_si_001.pdf]

Supporting Information for

Electrochemical Reduction Pathways from Goethite to Green Iron in Alkaline Solution with Silicate Additive

Divakar Arumugam, [a‡] *Tongxin Zhou,*[a‡] *Sathya Narayanan Jagadeesan,* [a‡] *Ranga Teja Pidathala,* [b] *Lihua Zhang,* [c] *AM Milinda Abeykoon,* [d] *Gihan Kwon,*[d] *Daniel Olds,*[d] *Badri Narayanan,* [b] *and Xiaowei Teng* [a]*

a Department of Chemical Engineering, Worcester Polytechnic Institute, 100 Institute Road, Worcester, MA 01609, United States
b Department of Mechanical Engineering, University of Louisville, 332 Eastern Pkwy, Louisville, Kentucky 40292, United States
c National Synchrotron Light Source II, Brookhaven National Laboratory, Upton, New York 11973, United States
d National Synchrotron Light Source II, Brookhaven National Laboratory, 743 Brookhaven Avenue, Upton, New York 11973, United States

* xteng@wpi.edu

Experimental Methods

Half-cell electrochemical measurements: Half-cell measurements were conducted in CH Instruments 660D/E electrochemical workstation. A three-electrode half-cell system was designed using a glassy carbon rotating-disk working electrode coated with the active material, a platinum wire counter electrode, and a mercury/mercury oxide reference electrode filled with 1 M NaOH solution. The ink was prepared by mixing the FeOOH active material and carbon black in 7:3 ratio in DI water at a concentration of 1 mg/mL. Then, 20 µg of the mixture was loaded on the working electrode, and 20 µL of 1 wt% Nafion 117 (Sigma Aldrich) was coated and vacuum dried on the top of the electrode material as a binder. A 100 mL plastic flask containing the 0.01 M NaOH electrolyte was used to conduct the linear sweep voltammetry (LSV) measurements, and the electrolyte was bubbled with Argon gas before and during the half-cell measurements. The LSV data were collected within the potential window of 0 V to -1.7 V (vs. Hg/HgO) with a sweep rate of 1 mV/s. Tafel slopes were calculated in the hydrogen evolution reaction (HER) region from the potential between -1.45 V and -1.55 V.

Operando **X-Ray Diffraction (XRD) measurements:** *Operando* XRD measurements were performed at beamline 28-ID-1 at the National Synchrotron Light Source – II in Brookhaven National Laboratory. A home-made acrylic cell sealed with an O-ring was used in the *Operando* XRD measurements. Six layers of cellulose-based filter paper were used to provide pressure on top of the working electrode for the experiment with 0.01 M NaOH/5000 ppm Na_2SiO_3 electrolyte. The ink slurry was prepared by mixing 70% of FeOOH active material and 30% of carbon black in a solvent mixture of 800 µL ethanol and 200 µL DI water at a concentration of 10 mg/mL along with 5 wt% Nafion 117 solution as the binder at a concentration of 30 µL/mL solvent. The working electrodes were prepared by drop-casting 200 µL of the slurry on a 5% Teflon-coated Toray carbon paper (Fuel Cell Earth) of an area of 1 cm^{-2} to give an active material loading of 1.4 mg cm^{-2}. The *Operando* XRD measurements were conducted using the working electrode mentioned above, a platinum wire counter electrode, and a silver/silver chloride reference electrode filled with 3 M KCl solution. Chronopotentiometry (CP) measurements were conducted from 0.2 V (vs. Hg/HgO) towards the negative potential (reduction) at a current density of 0.1 A g^{-1} in 0.01 M NaOH electrolytes with and without 5000 ppm of Na_2SiO_3. The XRD images were simultaneously captured in a 2D area detector during the electrochemical reduction. The wavelength of the synchrotron beam was 0.1665 Å. The synchrotron instrument parameters were calibrated using the CeO_2 standard. The phases from the XRD patterns were analyzed using the Rietveld refinement phase analysis method in the GSAS-II software. The patterns were re-plotted using Cu Kα radiation wavelength (λ = 1.5406 Å).

Operando **X-ray Absorption Spectroscopy (XAS) measurements:** *Operando* XAS measurements were done at beamline 6-BM for Materials Measurement at the National Synchrotron Light Source-II in Brookhaven National Laboratory. The home-made electrochemical cell mentioned before was used with six filter papers on top of the working electrode to provide support. The *Operando* XAS measurements were conducted using a

working electrode with 1.4 mg of active material (total loading of 2 mg containing 70% of FeOOH active material, 30% of carbon black) coated on 1 cm^{-2} of 5% Teflon-coated Toray carbon paper, a platinum wire counter electrode, and a silver/silver chloride reference electrode filled with 3 M KCl solution. Chronopotentiometry (CP) measurements were conducted from 0.2 V (vs. Hg/HgO) towards the negative potential (reduction) at a current density of 0.2 A g^{-1} in the 0.01 M NaOH electrolyte with 5000 ppm of Na_2SiO_3. The *Operando* XAS measurements were carried out in transmission mode at the Fe K-edge (7112 eV) simultaneously during the electrochemical reduction and the XAS spectra were collected ~ every 7 minutes. Fe metal foil and iron oxide powders (FeO, Fe_3O_4 and FeOOH) were used as references for X-ray energy calibration and data alignment. Athena software from the Demeter package was used to process and analyze XAS data.

Pulse-Relaxation Method to Measure the Diffusivity: After attaining open circuit potential (OCP) following the first charging cycle in 0.01 M NaOH solution (C) with different concentrations of silicates, a constant negative current pulse (I) of 0.25 A g^{-1} was applied for 15 seconds (Δt). The electrode material is then relaxed after the short-term current pulse. From the decay rate of the transient voltage (E) plotted against $t^{-1/2}$, the slope (k) was calculated. The chemical diffusivity (D) is proportional to $1/k^2$ by assuming initial occupancies of the electrochemical site on the electrode (y) are constant at different measuring conditions, where the term of (1-y) stands for available electrochemical sites in the electrode for the redox reaction (Equations S1 – S3).

$$\frac{E_0-E}{RT}=\frac{I\Delta t}{F^2C(1-y)\sqrt{\pi Dt}}$$ **Equation S1**

$$D=\frac{1}{k^2}\frac{1}{\pi}\left(\frac{RT*I\Delta t}{F^2C(1-y)}\right)^2$$ **Equation S2**

Where $$k=\frac{E_0-E}{\left(\frac{1}{\sqrt{t}}\right)}$$ **Equation S3**

Reactive molecular dynamics simulations: RMD simulations were performed using the widely recognized open-source package LAMMPS.[1-3] The electrolyte (0.01 M NaOH) was modeled using a computational supercell (12 nm × 12 nm × 12 nm) containing 15,000 water molecules and 3 NaOH molecules. We applied periodic boundary conditions in all directions. The desired number of silicate (SiO_3^{2-}) and Na^+ counterions were added at random locations in the supercell to achieve the prescribed concentration of the silicate additive. The interactions between Na, Si, O and H atoms were described by a reactive force field (ReaxFF) using parameters developed for $NaSiO_x$/water system,[4, 5] following our previous work on silicate/water systems.[6] Initial configurations were first equilibrated in an isothermal-isobaric (NPT) ensemble under ambient conditions for 0.5 ns. The resulting density of 1.05 g/mL[3] was in excellent agreement with experimental values. After equilibration, production runs were performed under ambient conditions for 1.5 ns in the canonical ensemble (NVT).

Density functional theory calculations: DFT calculations were performed within the framework of Hubbard corrected DFT theory (DFT +*U*) using the projected augmented plane wave method as implemented in VASP.[7-9] Exchange-correlation effects were treated with the Perdew-Burke-Ernzerhof (PBE) functionals under the generalized gradient approximation (GGA), using VASP's provided pseudopotentials: Na_pv (valence: $2p^6$ $3s^1$), Fe_pv ($3p^6$ $3d^6$ $4s^2$), Si ($3s^2$ $3p^2$), H (valence: $1s^1$), and O (valence: $2s^2$ $2p^4$).[8] A Hubbard correction (U = 6.5 eV and J = 0.5 eV) was applied to account for electron localization on Fe atoms, following the rotationally invariant DFT + U approach formulated by Liechtenstein et al.[10]

The computational supercell consisted of a (001) slab of $Fe(OH)_2$ and Fe_3O_4, built using a 6×4×1 and 1×2×1 unit cell, respectively, oriented with the surface normal along the [001] crystallographic direction. Periodic boundary conditions were imposed in all directions, and a vacuum gap of 23 Å was introduced along the surface normal to prevent interactions between periodic images. The plane wave energy cutoff was set to 520 eV, and a 1×1×1 Γ-centered Monkhorst-Pack k-grid for $Fe(OH)_2$ and 4×2×1 for Fe_3O_4 respectively was used to sample the Brillouin zone. Gaussian smearing with a width of 0.05 eV was applied. Atomic coordinates were optimized using the conjugate gradient method until atomic forces were less than 0.005 eV/Å. Long-range van der Waals interactions were included using the DFT-D3 method by Grimme with zero damping, as implemented in VASP.[11]

To compute the adsorption energies of $H_3SiO_4^-$ ions, the pristine $Fe(OH)_2$ and Fe_3O_4 slab was first relaxed. Then, $H_3SiO_4^-$ ions were introduced near the surface of the (001) slab, with Na^+ ions placed far from the surface to maintain charge neutrality. Multiple adsorption configurations were explored to identify the most energetically favorable one (i.e., the configuration with the most negative binding energy). For each configuration, the binding energy of the ion was calculated as $E_b = E_{ad} - E_{ion} - E_{slab}$, where E_{ad} is the total energy of the slab with the adsorbed ion, and E_{ion} and E_{slab} are the energies of the isolated ion and pristine slab, respectively.

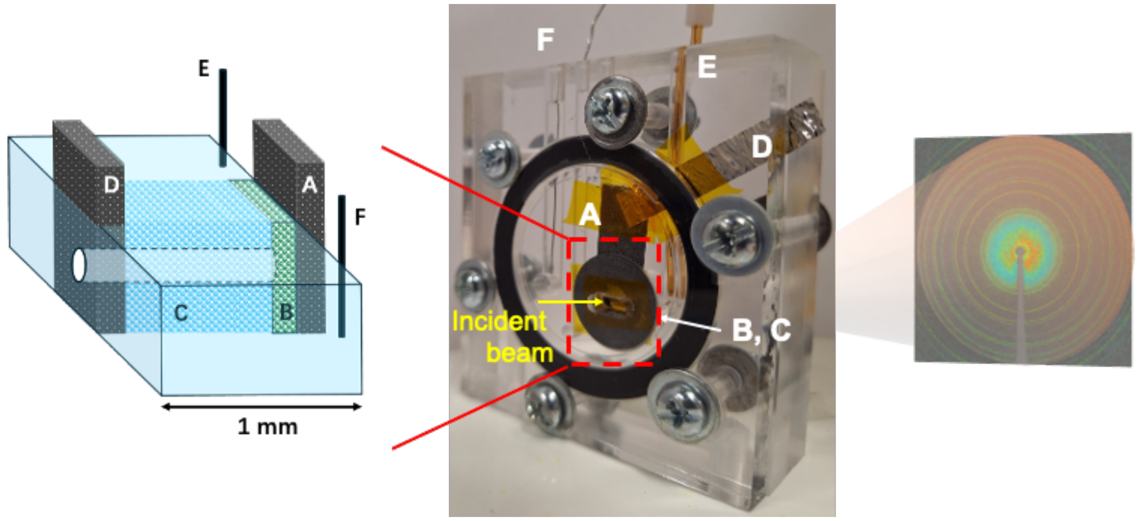

Figure S1. The electrolysis cell design. A: carbon paper current collector, B: active material working electrode (cathode), C: filter paper separator, D: carbon paper, E: Ag/AgCl reference electrode, and F: platinum wire counter electrode (anode)

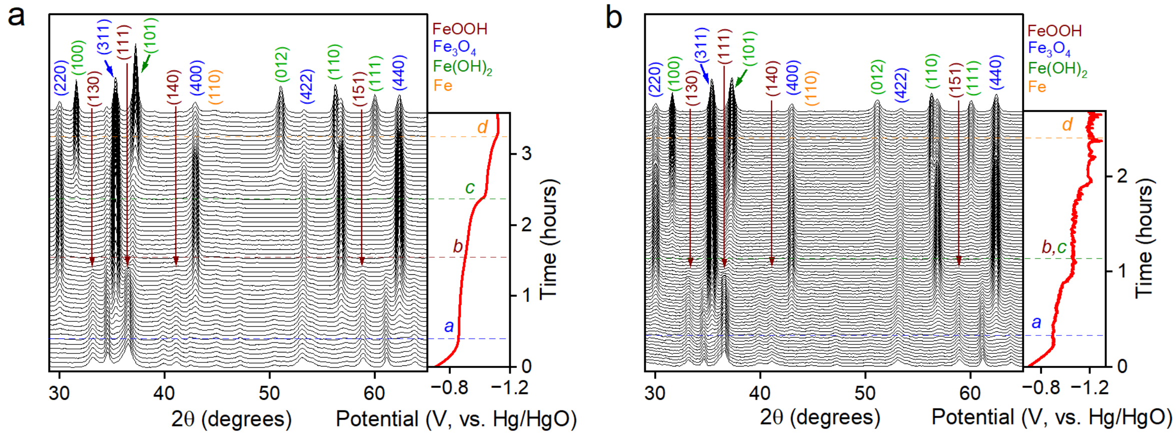

Figure S2. Stacked waterfall patterns of *Operando* XRD measurements in (a) 0.01M NaOH/5000 ppm Na_2SiO_3 and (b) 0.01M NaOH electrolytes acquired simultaneously with chronopotentiometry measurements at a current density of 0.1 A g^{-1}.

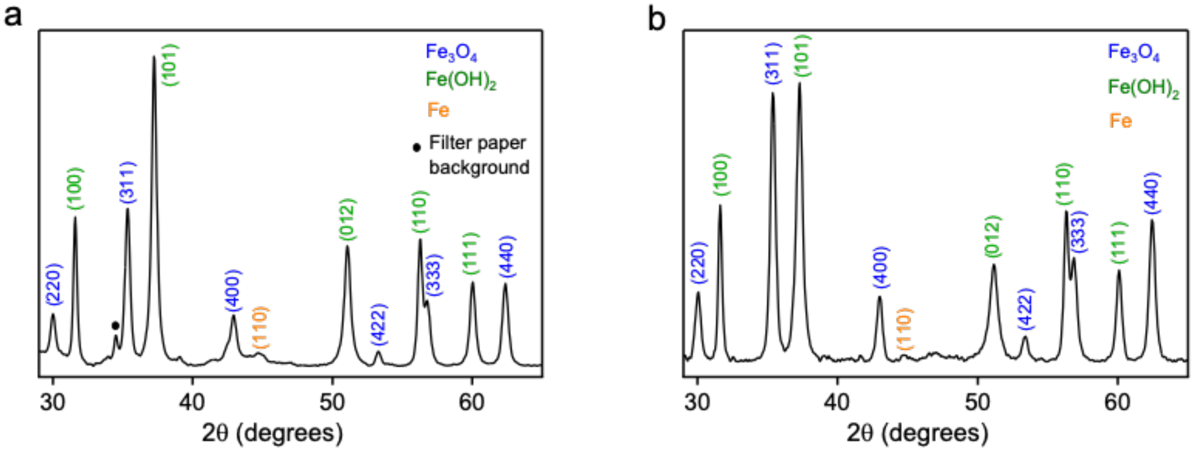

Figure S3. XRD patterns of electrochemical reduction products from (a) NaOH/silicate electrolytes and (b) NaOH electrolytes. The $Fe(OH)_2$ shows a stronger diffraction signal in silicate-containing electrolytes, leading to more Fe production.

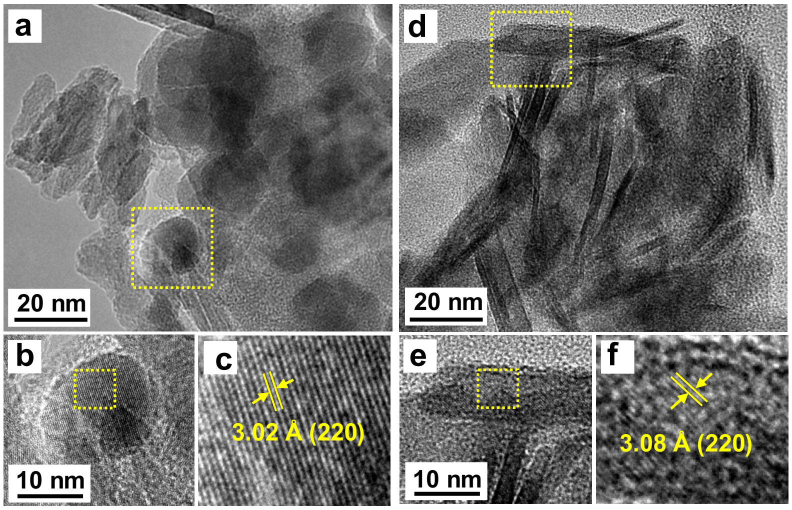

Figure S4. HR-TEM of electrochemical reduction products from (a-c) NaOH/silicate electrolytes and (d-f) NaOH electrolytes, showing (220) fringes lattice of Fe_3O_4.

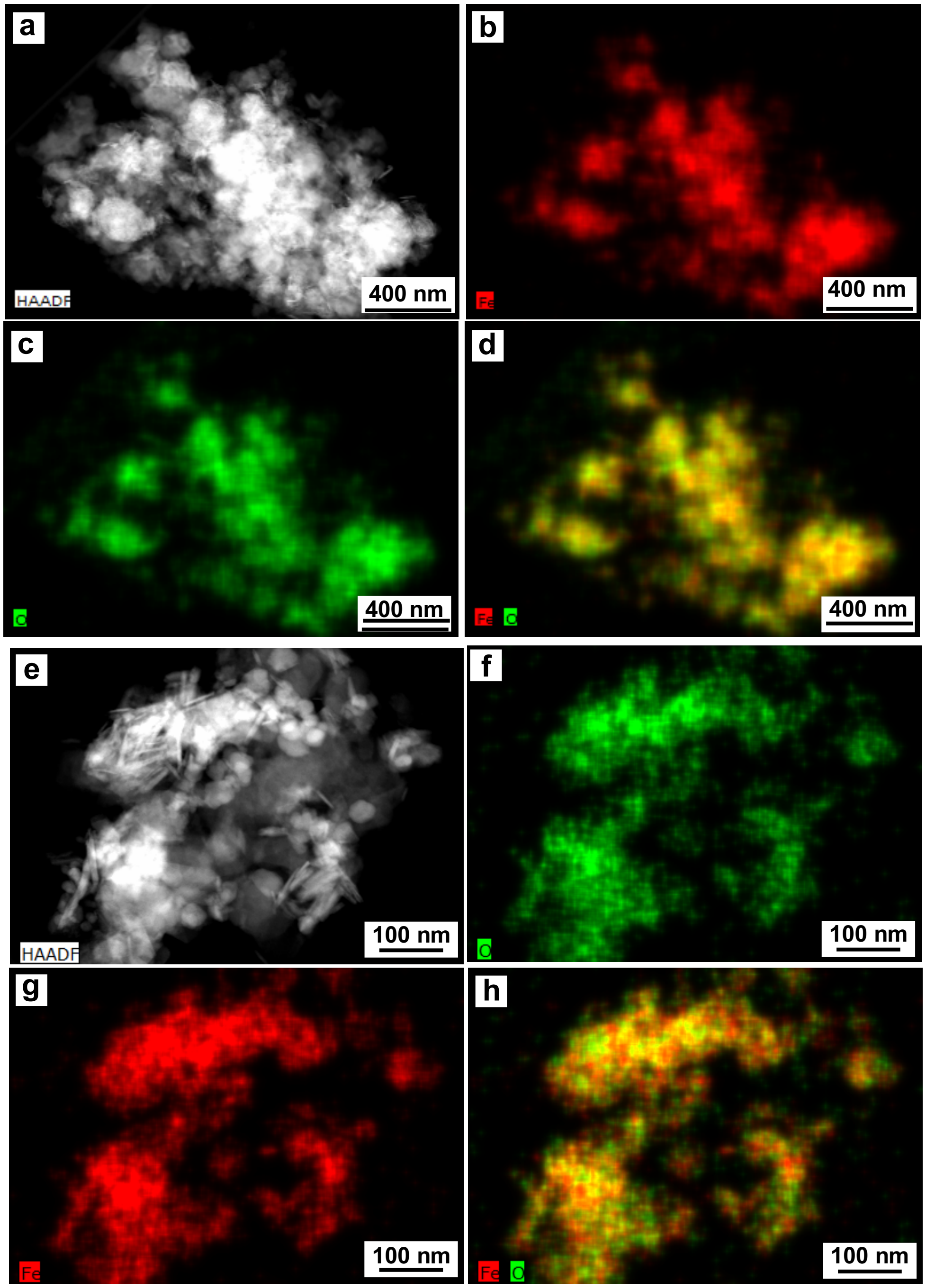

Figure S5. The STEM-EDS elemental mapping of final products prepared in (a-d) NaOH/silicate electrolyte and (e-h) NaOH electrolytes. The distributions of Fe and O are labeled in red and green, respectively. (d, f) the combined Fe and O mapping.

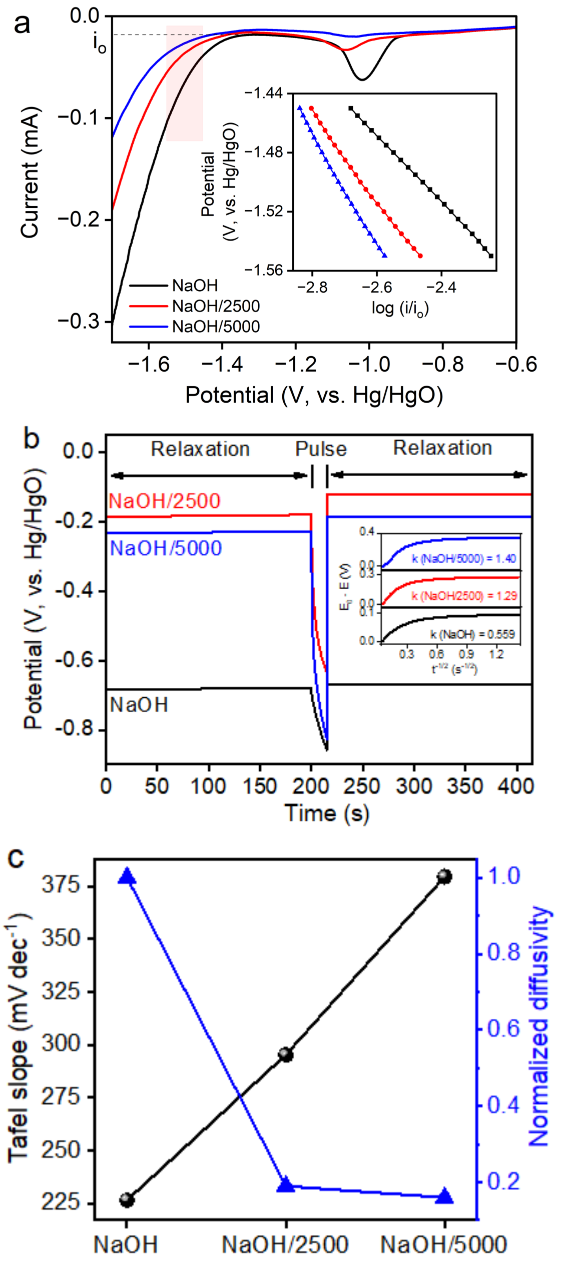

Figure S6. (a) Linear sweep voltammetry curves of FeOOH reduction in 0.01M NaOH, 0.01M NaOH/2500 ppm Na_2SiO_3 (NaOH/2500), and 0.01 M NaOH/5000 ppm Na_2SiO_3 (NaOH/5000) electrolytes at a scan rate of 1 mV s^{-1} with the inset of Tafel slopes, (b) current-pulse relaxation measurements, (c) summary of Tafel slopes and normalized diffusivities.

Reference:

(1) Plimpton, S. Fast Parallel Algorithms for Short-Range Molecular Dynamics. *J. Comput. Phys.* **1995**, *117* (1), 1-19.
(2) Aktulga, H. M.; Fogarty, J. C.; Pandit, S. A.; Grama, A. Y. Parallel reactive molecular dynamics: Numerical methods and algorithmic techniques. *Parallel Comput.* **2012**, *38* (4), 245-259.
(3) Thompson, A. P.; Aktulga, H. M.; Berger, R.; Bolintineanu, D. S.; Brown, W. M.; Crozier, P. S.; in 't Veld, P. J.; Kohlmeyer, A.; Moore, S. G.; Nguyen, T. D.; Shan, R.; Stevens, M. J.; Tranchida, J.; Trott, C.; Plimpton, S. J. LAMMPS - a flexible simulation tool for particle-based materials modeling at the atomic, meso, and continuum scales. *Comput. Phys. Commun.* **2022**, *271*, 108171.
(4) Hahn, S. H.; Rimsza, J.; Criscenti, L.; Sun, W.; Deng, L.; Du, J.; Liang, T.; Sinnott, S. B.; van Duin, A. C. T. Development of a ReaxFF Reactive Force Field for NaSiOx/Water Systems and Its Application to Sodium and Proton Self-Diffusion. *J. Phys. Chem. C* **2018**, *122* (34), 19613-19624.
(5) Hahn, S. H.; van Duin, A. C. T. Surface Reactivity and Leaching of a Sodium Silicate Glass under an Aqueous Environment: A ReaxFF Molecular Dynamics Study. *J. Phys. Chem. C* **2019**, *123* (25), 15606-15617.
(6) Jagadeesan, S. N.; Guo, F.; Pidathala, R. T.; Abeykoon, A. M. M.; Kwon, G.; Olds, D.; Narayanan, B.; Teng, X. Unlocking High Capacity and Reversible Alkaline Iron Redox Using Silicate-Sodium Hydroxide Hybrid Electrolytes. *ChemSusChem* **2024**, *n/a* (n/a), e202400050.
(7) Kresse, G.; Furthmüller, J. Efficient iterative schemes for ab initio total-energy calculations using a plane-wave basis set. *Phys. Rev. B* **1996**, *54* (16), 11169-11186.
(8) Perdew, J. P.; Burke, K.; Ernzerhof, M. Generalized Gradient Approximation Made Simple. *Phys. Rev. Lett.* **1996**, *77* (18), 3865-3868.
(9) Kresse, G.; Joubert, D. From ultrasoft pseudopotentials to the projector augmented-wave method. *Phys. Rev. B* **1999**, *59* (3), 1758-1775.
(10) Liechtenstein, A. I.; Anisimov, V. I.; Zaanen, J. Density-functional theory and strong interactions: Orbital ordering in Mott-Hubbard insulators. *Phys. Rev. B* **1995**, *52* (8), R5467-R5470.
(11) Grimme, S.; Ehrlich, S.; Goerigk, L. Effect of the damping function in dispersion corrected density functional theory. *J. Comput. Chem.* **2011**, *32* (7), 1456-1465.
